# The efficacy, biodistribution and safety of an inhibin DNA vaccine delivered by attenuated *Salmonella choleraesuis*

**DOI:** 10.1111/1751-7915.13029

**Published:** 2017-12-04

**Authors:** Wei‐zhen Chen, Ying‐mei Li, Xue Yu, Yue Li, Wen‐ke Li, Qing‐ling Wang, Ai‐xin Liang, Xiang Li, Li‐guo Yang, Li Han

**Affiliations:** ^1^ College of Veterinary Medicine Huazhong Agricultural University Wuhan 430070 China; ^2^ Tianjin Helaiente biological science and Technology Co., Ltd Tianjin 301709 China; ^3^ Key Laboratory of Agricultural Animal Genetics, Breeding and Reproduction of Ministry of Education College of Animal Science and Technology Huazhong Agricultural University Wuhan 430070 China; ^4^ State Key Laboratory of Agricultural Microbiology College of Veterinary Medicine Huazhong Agricultural University Wuhan 430070 China

## Abstract

DNA vaccines, the third‐generation vaccines, were extensively studied. The attenuated *Salmonella choleraesuis* (*S. choleraesuis*) was widely focused as a carrier to deliver DNA vaccines in the chromosome–plasmid balanced‐lethal system. The efficacy of inhibin DNA vaccine delivered by attenuated *S. choleraesuis* was proved in mice and cows in our previous studies. In this study, the efficacy of inhibin DNA vaccine was confirmed in rhesus monkeys. To further study the biodistribution and safety, the mice were immunized under laboratory conditions. The results of the rhesus monkeys showed the plasma IgA and IgG titres against inhibin were elevated, and the oestradiol (E_2_) and progesterone (P_4_) levels were increased with immunizing inhibin DNA vaccine. The biodistribution and safety assessment displayed the body weight, pathological change and haematology indexes where there is no significant difference between vaccinated mice and control. And the genomics analysis showed there was no integration of the inhibin gene into the mouse genome 2 months after immunization. This study indicated the inhibin DNA vaccine delivered by attenuated *S. choleraesuis* was safe. And this vaccine was a potential means to improve their reproductive traits in primates and other animals.

## Introduction

The double‐mutant *Salmonella choleraesuis* C500 strain with *asd* ^*−*^ (aspartate–semialdehyde dehydrogenase) and *crp* ^*−*^ (cAMP receptor protein) has been widely used as a carrier for DNA vaccines (Karpenko *et al*., 2012; Qiu *et al*., 2013). Although the pathogenicity of the strain is reduced, it retained its invasive capacity for carrying extraneous genes into the cell to elicit specific immune responses (Pathangey *et al*., 2009; Torres‐Escobar *et al*., 2010; Ashraf *et al*., 2011). The strain employs an Asd^+^ balanced‐lethal system in which the *asd* gene is a selective marker rather than an antibiotic resistance marker, thus avoiding the development of antibiotic resistance (Nakayama *et al*., 1988; Yang *et al*., 2010). Therefore, the system has been widely used in vaccine production (Bauer *et al*., 2005; Cazorla *et al*., 2008; Zhao *et al*., 2008). The safety of this system for delivering an extraneous gene has also been demonstrated (Liang *et al*., 2014).

As a third‐generation vaccine, DNA vaccines can elicit both humoral and cellular immune responses against extraneous genes (Pereira *et al*., 2014). Compared with other vaccine technologies, the advantages of DNA vaccines include flexible genetic design, no risk of infection, high reagent stability and a relatively low production cost in the microbial host (Liang *et al*., 2008). DNA vaccines have been widely used for various diseases, including avian influenza virus (Pan *et al*., 2009) and HIV (Tuomela *et al*., 2005). Immunization against the inhibin α‐subunit, which is the functional centre of the heterodimeric protein, increases the serum FSH concentration and, consequently, follicle development in mice (Medan *et al*., 2004a), goats (Wang *et al*., 2009), guinea pigs (Shi *et al*., 2000) and heifers (Akagi *et al*., 2002). Therefore, the inhibin α‐subunit may be a potential novel DNA vaccine target for enhancing ovulation in animals.

We previously demonstrated that immunization of mice with the inhibin gene delivered by the *asd* ^*−*^ */crp* ^*−*^ *S. choleraesuis* C500 strain increased the plasma anti‐inhibin titre (Han *et al*., 2014). And the previous study showed that the breeding rate of rhesus monkeys is low (Johnson *et al*.,1995; Lehman *et al*., 1994), and taking effective methods to improve their breeding is necessary. Therefore, this study aimed to study the effect of this vaccine on reproductive and immune function in rhesus monkeys. In addition, as the rhesus monkey is an endangered species, it is impossible to study the vaccine *in vivo*. Therefore, the vaccine distribution and biosafety were examined in mice. The results demonstrated that the chromosome–plasmid balanced‐lethal system is a safe and efficient approach for improving reproductive traits in primates. In summary, this study provides new ideas for the development of DNA vaccines and a foundation for the development of an inhibin DNA vaccine from the laboratory to clinical applications in primate.

## Results

### Ordinary clinical observations of experimental animals

During the experimental period, the immunized mice and rhesus monkeys exhibited healthy diets and normal stool patterns without any mortality. The animal weights increased gradually, and their general activities and mental statuses were consistent with good condition.

### Rhesus monkey plasma IgA and IgG titres

**Figure 1:**
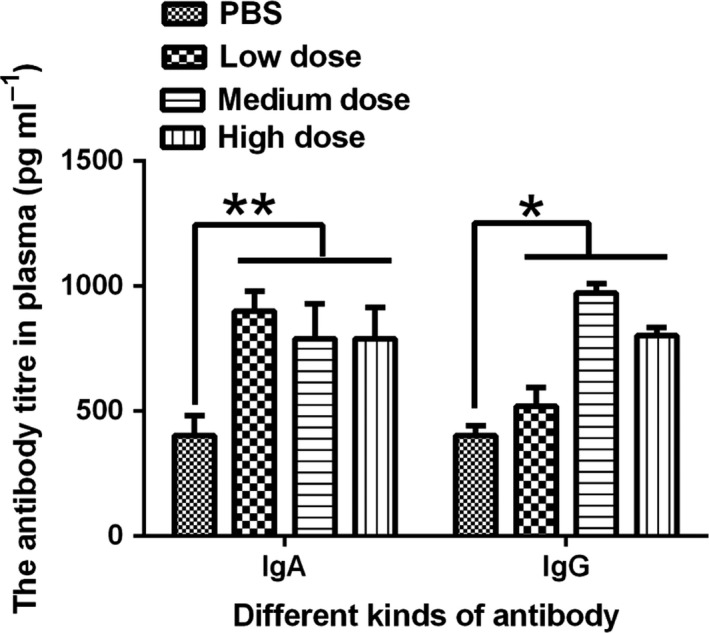
The anti‐inhibin IgA and anti‐inhibin IgG titres in rhesus monkey plasma at 28 days after immunization. Note: *P <* *0.05, and **P <* *0.01 were set as significant.

To study the efficacy of the *asd* ^*−*^ */crp* ^*−*^ C500/pVAX‐asd‐IS strain on the immune system, the antibody titre at 28 days in rhesus monkey after immunization was detected. The result showed that the IgA titres in the three dose groups were higher than those in the control group at 28 days (*P *<* *0.01) (Fig. 1). Furthermore, the IgG titre was significantly higher in the test groups than that in the control group at 28 days after immunization (*P *<* *0.05) (Fig. 1). However, there was no dosage‐dependent pattern. The result indicated that the inhibin vaccine can elicit obvious immune response in rhesus monkey.

### Plasma E_2_ and P_4_ concentrations in rhesus monkeys

**Figure 2:**
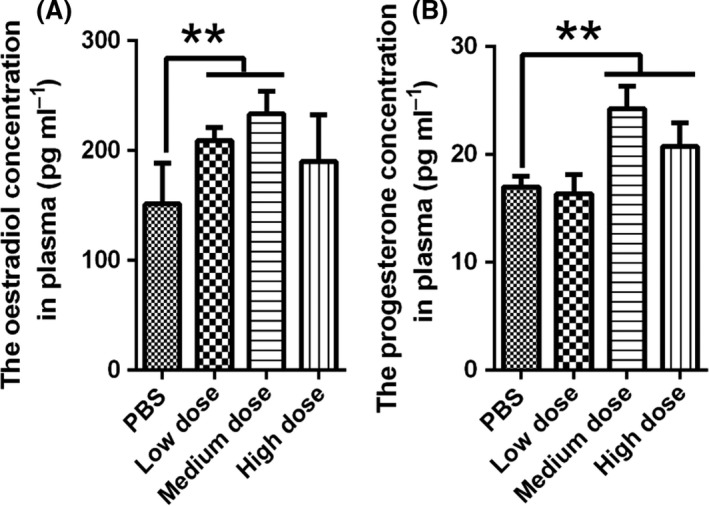
The oestradiol (A) and progesterone (B) concentrations in rhesus monkey plasma at 28 days after immunization. Note: **P <* *0.01 was set as significant.

To study the endocrine response elicited by recombinant inhibin vaccine in rhesus monkey, the E_2_ and P_4_ levels in plasma were detected by ELISA. Compared with the control group, the plasma E_2_ concentrations in the low and medium dosage groups were significantly higher at 28 days after immunization (*P *<* *0.01) (Fig. 2A). In addition, After immunization, the levels obviously increased, and the values were higher in the medium and high dosage groups than those in the control group at 28 days (*P *<* *0.01) (Fig. 2B). The result indicated that the medium dosage vaccine was the best suitable for increasing the hormone secretion.

### Strain distribution in mice

**Figure 3:**
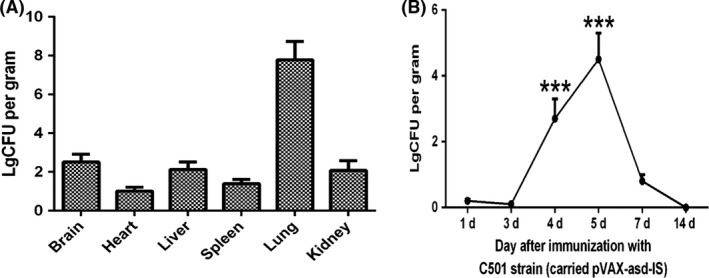
(A) The distribution of strain in tissue of mice at 5 days after immunization with C501 containing recombinant inhibin eukaryotic expression plasmid pVAX‐asd‐IS. (B) The distribution of strain in mice lung within 14 days after immunization with C501 strain. The value at 1 day was regarded as the control. Note: ***P <* *0.001 was set as significant.

To study the strain distribution in mice, the strain number in mice tissue was calculated. The result showed that no bacterial colonies were observed at 8 h after immunization. Bacterial colonies were first observed at 1 day after immunization in the groups immunized with C501 containing recombinant inhibin eukaryotic expression plasmid pVAX‐asd‐IS (Fig. 3B) and were mainly localized in the liver, lung, kidney, brain, spleen and heart and not the ovary with highest level in lung (Fig. 3A). Then, the strain distribution result in the lung was regarded as example and is shown in Fig. 3B, and the value at 1 day was regarded as the control. The bacterial colonies gradually increased from 4 days (*P *<* *0.001) and reached a maximum at 5 days (*P *<* *0.001). Thereafter, the bacterial colonies started to decrease at 7 days (*P *<* *0.05) and disappeared from all tissues by 14 days postimmunization. Organs from the control groups displayed no positive bacterial colony formation (data not shown).

### Blood physiology and biochemistry analysis in mice

**Figure 4:**
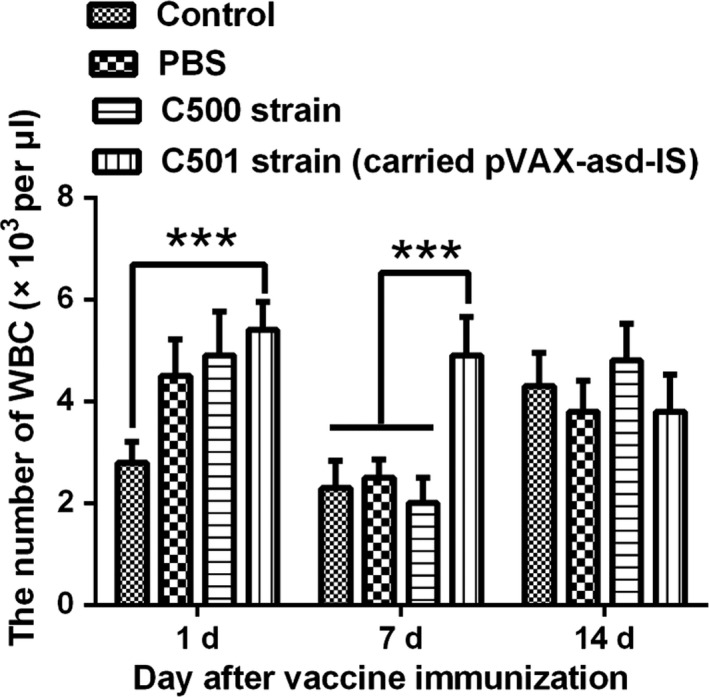
The number of WBC in mice blood postimmunization. 1 day: at 1 day postimmunization, 7 days: at 7 days postimmunization, 14 days: at 14 days postimmunization. Note: ***P <* *0.001 was set as significant.

WBC was higher in the test group than that in the control group at 1 and 7 days after immunization (*P *<* *0.001). However, there were no differences between the test and control groups at 14 days (Fig. 4). No changes in other physiological indicators were observed during the experimental period after vaccine immunization (*P *>* *0.05) (data not shown). Additionally, there were no differences in biochemical indicators between the test group and the control group (*P *>* *0.05) (data not shown).

### Effects of immunization on weight and organ pathological changes in mice

The weights of the heart, liver, spleen, lungs, kidneys, brain and ovary did not differ significantly between the test groups and the control group (*P *>* *0.05) (data not shown). Similarly, no significant abnormalities were observed in the pathological assay (data not shown).

### Gene integration of the recombinant plasmid in mice

**Figure 5:**
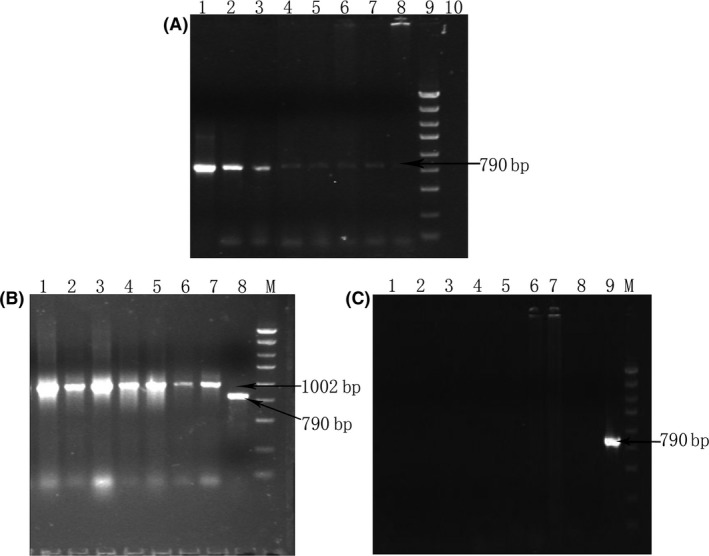
(A) The sensitivity of PCR for detection of recombinant inhibin eukaryotic expression plasmid (pVAX‐asd‐IS). Lane l–8: naive mice purified genomic DNA plus 10^10^, 10^9^, 10^7^, 10^5^, 10^4^, 10^3^, 10^2^, 10 copies of recombinant plasmid respectively; Lane 9: 5000 bp Marker; Lane 10: negative control. (B) PCR analysis of genomic DNA as template by GAPDH. Lane 1–7: heart, liver, spleen, lung, kidney, brain and ovary of female mice; Lane 8: 1 copy of recombinant plasmid (pVAX‐asd‐IS); M: 5000 bp Marker. (C) Representative PCR for recombinant plasmid (pVAX‐asd‐IS) in genomic DNA samples of female mice. Lane 1–7: heart, liver, spleen, lung, kidney, brain and ovary of female mice; Lane 8: water control; Lane 9: 1 copy of recombinant plasmid (pVAX‐asd‐IS); M: 5000 bp Maker.

This study explored the potential integration of the inhibin DNA gene into the host cell genome after oral immunization in mice. The assay sensitivity was approximately 1 plasmid copy/mg DNA (representing approximately 150,000 diploid cells) (Fig. 5A). The housekeeping gene GAPDH was amplified in all preparations as a control (Fig. 5B). There was no evidence of integration with a sensitivity of 1 plasmid copy/mg DNA (Fig. 5C).

## Discussion

Inhibin is secreted by ovary granular cells, and its mainly role is suppressing the secretion of follicle‐stimulating hormone (FSH) from the anterior pituitary gland (Robertson, 2012). Previous studies have shown that active or passive immunization with inhibin increases the secretion of FSH, which induces the development of follicles (Araki *et al*., 2000; Medan *et al*., 2003a,b). The selection of effective inhibin fragments is important for immunization success. Anderson *et al*. explored the immunization effect of inhibin in ewes and demonstrated that immunization with inhibin α (1–32) obviously increased follicular development (Anderson *et al*., 1998). Our laboratory has also demonstrated that immunization with inhibin α (1–32) significantly increases breeding in rats (Wang *et al*., 2012) and sheep (Han *et al*., 2008). Therefore, the present study used inhibin α (1–32).

Immunization with inhibin DNA vaccines has been studied in mono‐ and multi‐ovulatory animals (Medan *et al*., 2004a,b) but not primates. Therefore, the present study investigated the effect of the inhibin DNA vaccine on breeding of rhesus monkeys. In the present study, active immunization of rhesus monkeys with the inhibin DNA vaccine increased their IgA and IgG antibody titres. In support of this finding, a previous study demonstrated that immunization with an inhibin DNA vaccine improved the anti‐inhibin antibody titre in mice (Han *et al*., 2014). Liang *et al*. also indicated that mouse immunization with a somatostatin DNA vaccine increased the antisomatostatin antibody titre (Liang *et al*., 2014). These results indicate that inhibin DNA vaccine immunization can activate the immune system, resulting in the production of antibodies.

Medan *et al*. reported that active immunization against inhibin in cows increased the anti‐inhibin antibody titre, resulting in neutralization of endogenous inhibin and an increase in FSH that promoted follicular development, ovulation and corpora luteum formation (Medan *et al*., 2006). In the present study, E_2_ and P_4_ levels were significantly higher in the immunized groups than those in the control group. This result is in accordance with the results of a study by Sasaki in which he used an inhibin DNA vaccine to immunize goats and observed increasing in E_2_ and P_4_ levels (Sasaki *et al*., 2006). Mei *et al*. also demonstrated that immunization against inhibin in Holstein Heifers promoted the secretion of E_2_ and P_4_ (Mei *et al*., 2009). The increasing in E_2_ level potentially reflects an increasing in the number of preovulatory follicles, whereas the increasing in P_4_ may be related to an increasing in the number of corpus lutea (Medan *et al*., 2004b). In summary, the present results indicate that immunization with the inhibin DNA vaccine is an effective method for inducing ovulation in primates. Among the three dosages evaluated in the present study, the medium dosage was most effective in rhesus monkeys.

The present study indicated that the novel inhibin vaccine could increase the reproduction of monkey. Many previous studies have indicated that immunization with hormone purified from follicular fluid (Sewani‐rusike *et al*., 2000) could obviously stimulate follicular development (Wang *et al*., 2012) and enhance superovulation (Li *et al*., 2011). However, in comparison with hormone treatment, there is no need for preparation and purification of hormone which are time‐consuming and labour‐intensive. In addition, there are also no further plasmid extraction and purification steps compared with naked DNA vaccines, which reduced cost and labour considerably. Therefore, the DNA vaccine was an effective and economical biotechnology to improve animal fertility.

The present study utilized attenuated *S. choleraesuis* to deliver the inhibin DNA vaccine. In clinical, vaccine safety is an important factor that can inhibit its application. And there are guidance documents (FDA, 2007; WHO, 2007) for the clinical use of DNA vaccines which indicated that the biosafety of the vaccine relates to toxicological, genetic and environmental effects. After immunization, WBC in the test group was higher than that in the control group at 1 and 7 days. However, WBC recovered to normal at 14 days postimmunization. The increasing values indicate that an inflammation response occurred but disappeared at 14 days postimmunization. A previous study reported that the DNA vaccine induced an inflammation response that resolved after 6 months (Liu *et al*., 2008; Liang *et al*., 2014). Furthermore, the other physiological and biochemical blood indicators did not change significantly after immunization with the inhibin DNA vaccine.

The strain distribution analysis demonstrated that the bacteria had completely vanished from mouse organs at 14 days after vaccine immunization. These findings are consistent with a previous report (Mizuno *et al*., 2007) in which that the vaccination strain was isolated from the heart, blood, liver and spleen of mice at 1 day after immunization and was eliminated at the end of the study. The results also showed that the inhibin gene vaccine had no effect on organ weights or histopathological changes. Overall, the inhibin DNA vaccine had no adverse or toxic effects on various organ systems and tissues. These findings indicate that the attenuated *asd* ^*−*^ */crp* ^*−*^ *S. choleraesuis* C500 strain is safe for use as a DNA vaccine vehicle in mice.

The potential of the DNA vaccine to integrate into the host cell genome is the primary safety concern (Ledwith *et al*., 2000; Wang *et al*., 2004; Medjitna *et al*., 2006). The frequency of gene integration is affected by several factors, including the plasmid sequence, the presence of chi‐like elements (Manam *et al*., 2000), Alu segments (Rüdiger *et al*., 1995) and minisatellite regions (Krowczynska, 1990). However, plasmid DNA integration is not simple, and the mammalian genome appears to possess a mechanism to protect its own integrity (Scrable and Stambrook, 1999). In the present study, no evidence of integration after inhibin DNA vaccine immunization was observed in any of the investigated organs, suggesting the safety of this vaccine. Ledwith *et al*. evaluated different plasmid constructs and suggested that the risk of plasmid DNA vaccine integration following intramuscular inoculation is negligible (Ledwith *et al*., 2000). These results indicate that the inhibin DNA vaccine is safe for use in clinical applications.

## Conclusions

The present study demonstrates that the inhibin gene vaccine can activate an immune response and increase hormone secretion in rhesus monkeys. The results also indicated that delivery of the inhibin gene vaccine by the attenuated *asd* ^*−*^ */crp* ^*−*^ *S. choleraesuis* C500 strain is safe for clinical applications. The present study contributes to the application of DNA vaccines and lays a foundation for the use of the inhibin DNA vaccine in the clinic. This study also provides new ideas for the application of gene vaccines in primates.

## Experimental procedures

### Identification of the recombinant plasmid

The recombinant inhibin eukaryotic expression plasmid pVAX‐asd‐IS (containing inhibin fusion gene) was constructed in our laboratory as previously described (Han *et al*., 2008; Liu *et al*., 2016) and used here. The attenuated *asd* ^*−*^ */crp* ^*−*^ *S. choleraesuis* C500 strain was kindly provided by Professor Ai‐zhen Guo of Huazhong Agricultural University.

Bacteria transformed by electroporation with the pVAX‐asd‐IS plasmid and pVAX‐asd plasmid were cultured on Luria–Bertani (LB) medium. Ten bacterial colonies were randomly picked from each plate, and the plasmid DNA was extracted using a kit (TaKaRa, Dalian, China) according to the manufacturer's instructions. Plasmid DNA was digested with the HindIII and EcoRI restriction enzymes and analysed by agarose gel electrophoresis to identify the gene fragment. In addition, the potency of the vaccine *in vitro* expression test has been conducted in our previous experiment. The qPCR and Western blot were performed to detect the mRNA level and protein expression level of vaccine in HeLa cells (Han *et al*., 2008, 2014). Besides, the effect of hormone secretion on mouse, rat and buffaloes (Wang *et al*., 2012; Han *et al*., 2014; Liu *et al*., 2016) was also studied. All of those results showed that the vaccine could significantly increase reproduction.

### Preparation of the strain for vaccination

The strains carrying pVAX‐asd‐IS and pVAX‐asd were inoculated on LB medium, whereas the wild‐type strain was inoculated on LB medium with the addition of DAP (50 μg^−1^ ml). The bacteria were cultured at 37°C and shaking overnight at 200 r.p.m. for 40 generations to detect its stability, harvested by centrifugation at 6000 r.p.m. for 5 min, washed with phosphate‐buffered saline (PBS) and re‐suspended in a minimal amount of PBS. Besides, in our previous study, the housekeeping gene (invA) of the strain was tested by PCR to detect its stability (Zhen *et al*., 2009). The bacteria were then quantified by determining the number of colony‐forming units (CFU) in triplicate, and the values were expressed as CFU^−1^mL.

### Experimental animal

Two hundred female specific‐pathogen‐free (SPF) Kunming mice (37 days old) were purchased from the Hubei Center for Disease Control and Prevention (Wuhan, China). Twelve healthy female Chinese rhesus monkeys (4–5 years old) were chosen for the immunization assay. All animals were housed in a room with stable temperature (25°C) and a 12 h/12 h light/dark cycle with food and water. Abnormal behaviour was recorded during the experimental period. Before oral immunization, all mice were deprived of food for 12 h and water for 4 h. Thereafter, the mice were administered 100 μl of 7.5% sodium bicarbonate (NaHCO_3_) by orogastric gavage to neutralize gastric acid. All experiments involving animals were performed according to the National Institutes of Health Guide for the Care and Use of Laboratory Animals. All protocols were approved the by Ethics Committee of Huazhong Agricultural University, China.

### Rhesus monkey immunization schedule and sample collection

To detect the efficacy of the inhibin DNA vaccine on the immune and endocrine system, 12 rhesus monkeys were randomly allocated to four groups (n = 3). Compared with oral immunization, the vaccine intramuscular injection is easier to be conducted, and it is the least to induce the monkey stress which has the little effect on monkey. Therefore, the monkey received the vaccine by intramuscular injection. Before immunization, the animals received local anaesthesia with 300 μl of procaine. The control group received 1.0 ml PBS by intramuscular injection, whereas the test groups were immunized with 0.2 × 10^10^ CFU (low dose), 0.6 × 10^10^ CFU (medium dose) or 1.0 × 10^10^ CFU (high dose) of *asd* ^*−*^ */crp* ^*−*^ C500/pVAX‐asd‐IS strain by intramuscular injection. The booster immunization was conducted with the same dosage at a 14 days of interval. The blood samples were collected in heparinized tubes at 28 days after first immunization. Thereafter, the samples were centrifuged at 3200 r.p.m. for 10 min, and the plasma was stored at *−*20°C until further study.

### Mice immunization schedule and sample collection

To explore the distribution of the attenuated *asd* ^*−*^ */crp* ^*−*^ *S. choleraesuis* C500 strain and blood toxicity in mice after vaccine immunization, one hundred inbred mice were equally divided (n = 25) into four groups. The two control groups received 200 μl of PBS only or 2 × 10^10^ CFU of *asd* ^*−*^ */crp* ^*−*^ C500/pVAX‐asd, whereas the test group was immunized with 2 × 10^10^ CFU of *asd* ^*−*^ */crp* ^*−*^ C500/pVAX‐asd‐IS by intragastric administration. The other group was a control group without any treatment. Food and water supplies were reassessed 30 min after immunization. After immunization, the mice were killed to collect blood samples through the eyeball vein to study blood toxicity at 1, 7 and 14 days. The heart, liver, spleen, lung, kidney, ovary and brain were extracted under sterile conditions at 8 h and 1, 3, 4, 5, 7, 10 and 14 days after immunization from three mice at each time point to explore the strain distribution. The process was repeated at least twice.

To study gene integration, tissue weight and pathological changes in mice after vaccine immunization, one hundred inbred mice were equally divided (n = 20) into five groups. The control groups received 200 μl of PBS only or 2 × 10^10^ CFU of the C500 strain, whereas the test groups received 2 × 10^8^, 2 × 10^9^ or 2 × 10^10^ CFU of the *asd* ^*−*^ */crp* ^*−*^ C500/pVAX‐asd‐IS strain by intragastric administration. Food and water supplies were reassessed 30 min after immunization. All mice received two booster doses at 14 days of interval for a total of three immunizations. Seven days after immunization, a subset of the mice was killed, and the heart, liver, spleen, lung, kidney, ovary and brain were collected for weighing and histopathological analysis. We chose those organs because they were relate to metabolize, reproduction and vital sign to study the effect of vaccine. The remaining mice were raised for 2 months for gene integration analyses. The process was repeated at least twice.

### Detection of plasma IgA and IgG titres

The plasma immunoglobulin A (IgA) and immunoglobulin G (IgG) titres were detected by enzyme‐linked immunosorbent assay (ELISA). The protocol was performed according to the previously described methods (Wang *et al*., 2012). Briefly, synthetic inhibin fusion protein was coated onto a 96‐well plate at 4°C overnight. After washing, the antigen was blocked with 5% BSA. Then, plasma was added to every well, and every sample was detected in triplicate. Positive and negative controls were also performed. Next, HRP‐conjugated rabbit anti‐human IgA and IgG secondary antibodies (1:2000 dilution; Wuhan Yide Biological Technology, Wuhan, China) were added to the plate followed by the substrate solution. The reactions were terminated by the addition of 2 M sulfuric acid, and the absorbance values were detected at OD_450_. A P/N value > 2 was the optimal dilution ratio; P represents the OD_450_ value of the plasma to be tested; and N represents OD_450_ value of the plasma from the negative control.

### Detection of plasma E_2_ and P_4_ concentrations

Plasma E_2_ and P_4_ concentrations were measured by a ^125^I‐labelled RIA kit (Beijing North Institute of Biological Technology, Beijing, China). The assay was performed according to the manufacturer's instructions, and every sample was detected in triplicate. The assay sensitivity was 2 pg^−1^mL for E_2_ and 0.2 ng^−1^mL for P_4_. The intra‐ and intercoefficient variations were < 10% and < 15% for both kits.

### Strain distribution detection

After the strains from the heart, liver, spleen, lung, kidney, ovary and brain were isolated under aseptic conditions, the bacteria were homogenized and diluted with 0.5 ml of PBS. Then, 100 μl of each dilution was plated on MacConkey agar (Hangzhou Microbial Reagent, Hangzhou, China) at 37°C and incubated overnight. After positive identification, the number of colonies was calculated using the viable plate count method. The experiment was repeated in triplicate.

### Blood toxicity detection

After the blood was collected, anticoagulated blood (50 μl) was analysed by physiological testing, and serum was sent to the Seventh People's Hospital of Wuhan, China, for biochemical testing. Blood physiological tests were performed to identify the following eight indicators: white blood cell count (WBC), red blood cell count (RBC), haemoglobin (HGB), haematocrit (HCT), mean corpuscular volume (MCV), mean corpuscular haemoglobin (MCH), mean corpuscular haemoglobin concentration (MCHC) and platelet count (PLT). The blood biochemical tests included six indicators: aminotransferase (ALT), aspartate aminotransferase (AST), total protein (TP), albumin (ALB), globulin (GLO) and creatinine (CREA).

### Tissue weight and histopathological changes detection

After weighing, the tissues were fixed with 4% (w/v) paraformaldehyde (Boster, Wuhan, China) and embedded in paraffin. Three non‐adjacent sections (4 μm) were selected from each tissue at an interval of not less than 120 μm and stained with haematoxylin–eosin (HE). The sections were examined blindly by three independent observers for possible histopathological changes.

### Detection of inhibin DNA vaccine gene integration in mice

To analyse the PCR sensitivity, the recombinant plasmid was 10‐fold serially diluted 10 times. Then, 0.1 μg of plasmid was added to 0.3 μg of genomic DNA from mice that were not immunized with the recombinant plasmid, leading to plasmid concentrations from 10^0^ to 10^10^. The primer sequences, which were designed using Primer version 5.0, were TGCTGGATATCTGCAGAATTCCCT (sense) and CTTCTCGAGATCTGTGGCAGTCGG (antisense). PCR was performed using ddH_2_O as the negative control. The reaction system had a volume of 20 μl and included 2 μl of DNA template, 0.5 μl of primer (sense and antisense), 2.9 μl of Mix (Beijing TransGen Biotech, Beijing, China) and 14.1 μl of ddH_2_O. The reaction was performed under the following conditions: 94 °C for 10 min, followed by 40 cycles of 94 °C for 30 s, 60 °C for 45 s and 72°C for 45 s and a final elongation at 72 °C for 10 min.

The mouse GAPDH gene was detected by PCR to determine its integrity and conservation. The primer sequences were designed using Primer version 5.0 and were AGCCTCGTCCCGTAGACAAA ATGGT (sense) and GTGGGTGGTCCAGGGTTTCTTACTC (antisense). The reaction system had a volume of 20 μl and included 0.4 μl of DNA template, 0.5 μl of primer (sense and antisense), 2.9 μl of Mix and 15.7 μl of ddH_2_O. The reaction was performed according to the above method.

The DNA from the tissue samples was extracted with a kit (TIANGEN Biotech, Beijing, China) according to the manufacturer's instruction. The possibility of DNA integration into the mouse genome was analysed by PCR. The detection method was in accordance with the procedure of PCR sensitivity. Plasmid DNA was used as a positive control, and ddH_2_O was used as a negative control.

### Statistical analysis

All results are expressed as the mean ± SD. The data were analysed by ANOVA (spss, version 17.0; Chicago, IL, USA). Figures were prepared using GraphPad Prism 6, and *P <* *0.05, **P <* *0.01 and ***P <* *0.001 were considered statistically significant.

## Conflict of interest

None declared.
